# Epidemiology and survival outcomes of lip, oral cavity, and oropharyngeal squamous cell carcinoma in a southeast Brazilian population

**DOI:** 10.4317/medoral.25147

**Published:** 2022-04-03

**Authors:** Brendo Vinícius Rodrigues Louredo, Pablo Agustin Vargas, Maria Eduarda Pérez-de-Oliveira, Márcio Ajudarte Lopes, Luiz Paulo Kowalski, Maria Paula Curado

**Affiliations:** 1 DDS, MSc, PhD student. Department of Oral Diagnosis, Oral Pathology Area, Piracicaba Dental School, State University of Campinas (UNICAMP), Piracicaba, SP, Brazil; 2 DDS, PhD, FRCPath, Professor. Department of Oral Diagnosis, Oral Pathology Area, Piracicaba Dental School, State University of Campinas (UNICAMP), Piracicaba, SP, Brazil; 3 DDS, PhD, Professor. Department of Oral Diagnosis, Oral Semiology Area, Piracicaba Dental School, State University of Campinas (UNICAMP), Piracicaba, SP, Brazil; 4 MD, PhD. Department of Head and Neck Surgery, São Paulo State Cancer Institute (ICESP), School of Medicine, University of São Paulo (USP), São Paulo, SP, Brazil; 5 MD, PhD. Group of Epidemiology and Statistics on Cancer, International Research Center, A. C. Camargo Cancer Center (ACCCC), São Paulo, SP, Brazil

## Abstract

**Background:**

Lip, oral cavity, and oropharyngeal squamous cell carcinoma (SCC) represent a major health problem in the global scenario. In South America, the highest incidence rates are seen in Brazil. Therefore, the epidemiological and clinical profile and survival outcomes of lip, oral cavity, and oropharyngeal SCC was studied in São Paulo State, Brazil.

**Material and Methods:**

The clinicopathological data of 12,099 patients with lip, oral cavity, and oropharyngeal SCC were obtained from hospital cancer registries of the Fundação Oncocentro de São Paulo, Brazil (2010–2015). Survival rates and other analyses were performed using SPSS software.

**Results:**

A clear male predominance was observed, particularly for patients with oropharyngeal SCC (88.3%). The average age of patients was higher for lip cases (65 ± 13.5 years) compared to other sites. The schooling level was low for most patients, especially in lip cases (87.9%). Most of the patients with oral cavity (71.8%) and oropharyngeal (86.3%) SCC had advanced-stage (III–IV) disease. However, the majority of lip cases (83.3%) were at an early stage (I–II). Surgical excision was the main treatment for lip (72%) and oral cavity SCC (23.5%), and chemoradiotherapy was the main treatment for oropharyngeal SCC (40.2%). The 5-year overall survival (OS) for patients with lip, oral cavity, and oropharyngeal SCC were 66.3, 30.9, and 22.6%, respectively. Multivariate analysis revealed that the determinants of OS were different for lip, oral cavity, and oropharyngeal SCC, except for those at the clinical stage, which was an independent predictor for all sites.

**Conclusions:**

OS-independent determinants varied according to the affected site. Oral cavity and oropharyngeal SCC presented worse survival rates than those for lip SCC.

** Key words:**Squamous cell carcinoma of head and neck, lip neoplasms, mouth neoplasms, oropharyngeal neoplasms, survival analysis.

## Introduction

Oral cancer, including lip cancer, is one of the most common cancers around the world, falling within the top ten cancers in several countries, with an estimated 377,713 new cases in 2020. When analysed together with the oropharynx, these two locations comprise approximately 476,125 new cases, accounting for 2.5% of all cancer cases and 225,900 deaths (177,757 deaths for oral cancer and 48,143 deaths for oropharyngeal cancer) (1,2).

In 2020, the estimated age-standardised rates of oral cancer were 6.0 and 2.3 per 100,000 in men and women, respectively, whereas for oropharyngeal cancer, they were 1.8 and 0.4 per 100,000 in men and women, respectively (2). Most patients diagnosed with oral cavity and oropharyngeal cancers report a previous history of smoking and alcohol consumption, which are well recognised risk factors (3). Additionally, human papillomavirus (HPV) infection has been associated with the development of a distinct subset of head and neck squamous cell carcinomas (SCC), particularly in the oropharynx (4), and ultraviolet radiation from sunlight exposure for lip SCC (2).

The incidence of oral and oropharyngeal cancer in Central and South America is not homogenous, and the highest rates are seen in Brazil, particularly for males, and are up to three-times higher than in other Central and South American countries (5). The Fundação Oncocentro de São Paulo (FOSP) is a Brazilian public database that collects data from all hospitals that perform cancer treatment in São Paulo State, and it is updated every three months. The epidemiological and clinical profile and survival outcomes of the lip, oral cavity, and oropharyngeal SCC were assessed in the São Paulo State, Brazil, from a FOSP database (2010-2015).

## Material and Methods

- Sample

This is a retrospective cross-sectional study using secondary data. Data of patients with histopathological diagnosis of primary lip (International Classification of Diseases for Oncology [ICD-O-03]: C00), oral cavity (ICD-O-3: C02, C03, C04, C05 [except C05.1 and C05.2] and C06), and oropharyngeal (ICD-O-3: C01, C05.1, C05.2, C09, and C10) cancers in São Paulo State were obtained from hospital cancer registries in the FOSP database from January 2010 to December 2015 (available at: http://www.fosp.saude.sp.gov.br/publicacoes/downloadarquivos, accessed 15 January 2021). The following morphological codes: 8051/3, 8052/3, 8070/3, 8071/3, 8072/3, 8073/3, 8074/3, 8075/3, 8076/3, 8078/3, 8082/3, 8083/3, and 8084/3 used for lip, oral cavity, and oropharyngeal SCC were considered for analysis.

- Data collect

São Paulo State has 17 Health Regional Departments (HRDs): São Paulo, Araçatuba, Araraquara, Baixada Santista, Barretos, Bauru, Campinas, Franca, Marília, Piracicaba, Presidente Prudente, Registro, Ribeirão Preto, São João da Boa Vista, São José do Rio Preto, Sorocaba, and Taubaté. The following variables were collected: HRD, period of diagnosis, gender, age group, schooling level, primary tumour site, previous diagnosis and treatment, diagnosis origin, clinical stage (TNM: I-II and III-IV), time between diagnosis and treatment, cancer treatment, and patients’ status (alive or died).

- Statistical analysis

The qualitative and quantitative data were presented descriptively, and missing values were excluded from the analysis, with only valid percentages being considered. An association analysis between demographic and clinicopathological variables with tumour site was performed using the Chi-square test. All lip, oral cavity, and oropharyngeal SCC cases that reported the patients’ follow-up and status were included for survival analysis. The Kaplan-Meier method was used to estimate survival rates, and the difference between survival curves was investigated by using the Log-Rank univariate test. The univariate Cox proportional hazard regression model was employed to identify potential prognostic factors. A multivariate Cox regression model was created using all variables that achieved a *p-value* ≤ 0.20. Data analyses were performed with SPSS software version 22.0 (IBM Corporation, Armonk, NY, USA), and a *p-value* ≤ 0.05 was considered statistically significant.

## Results

**Figure 1:**
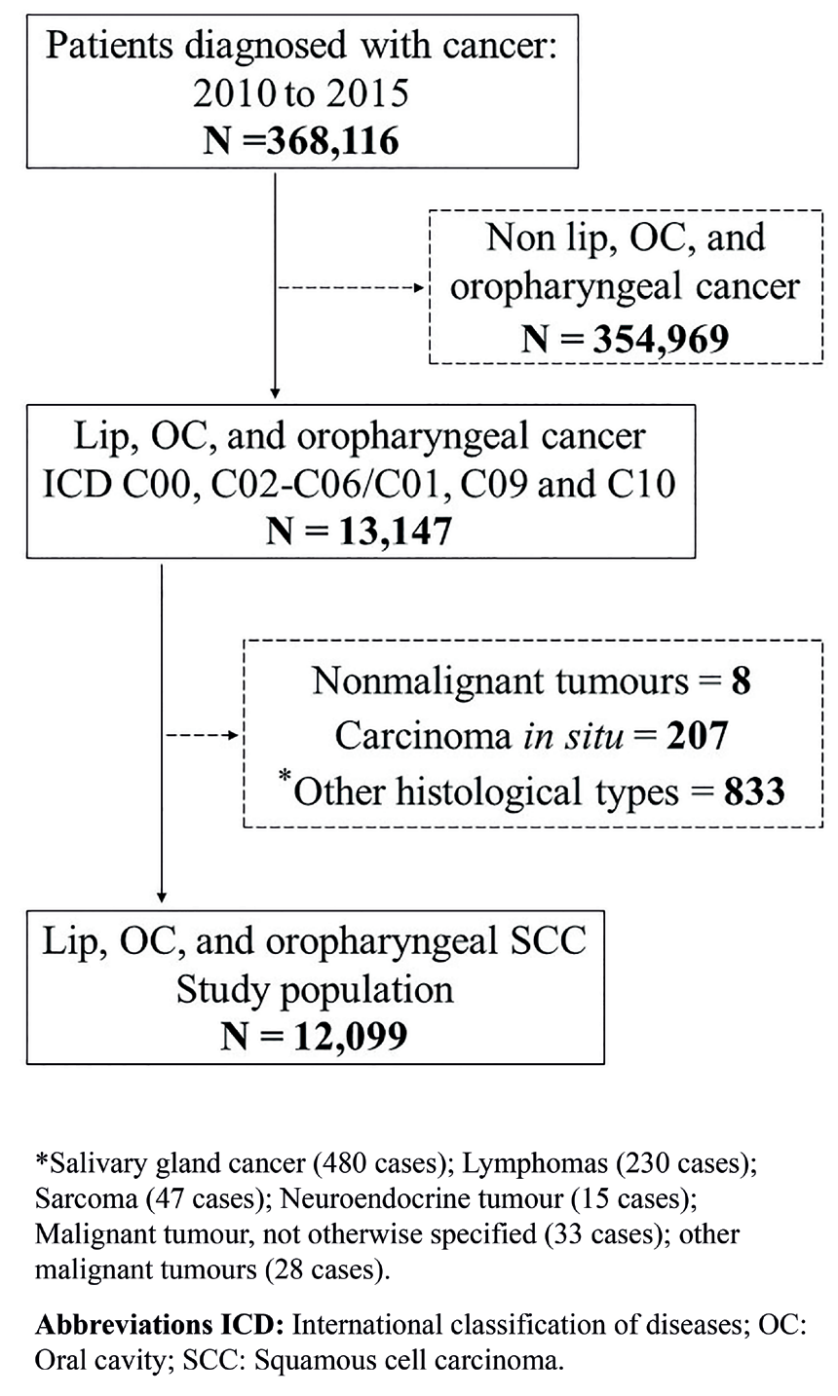
Flow diagram of selection of study sample.

**Figure 2:**
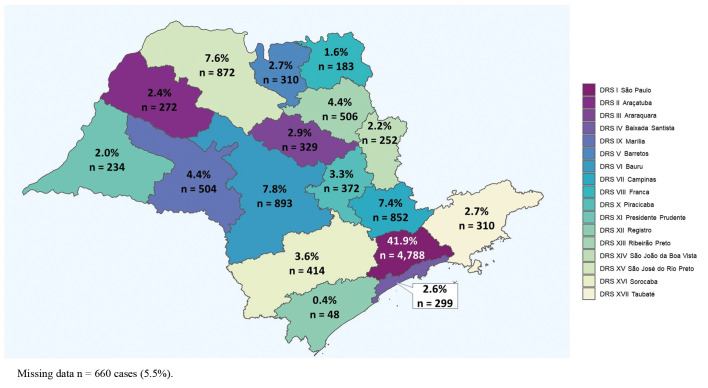
Distribution of lip, oral cavity, and oropharyngeal squamous cell carcinoma diagnosed between 2010 and 2015 according to 17 Health Regional Departments of São Paulo State.

The data collected from 76 hospital cancer registries (HCRs) of the São Paulo State found a total of 368,116 cancer cases in the period between 2010 and 2015. Of these, 12,099 patients were diagnosed with lip, oral cavity, and oropharyngeal SCC (Fig. 1). Fig. 2 shows the distribution of all cases according to the 17 HRDs in São Paulo State. The demographic and clinicopathological features of the 12,099 cases of lip, oral cavity, and oropharyngeal SCC are summarised in Table 1.

- Lip SCC

About 73.3% (732 cases) of 998 patients with lip SCC were male, with a male-to-female ratio of 2.8:1. Regarding schooling level, most individuals (87.9%; 717 cases) had less than or equal to 8 years of formal education. The patients' ages ranged from 22 to 104 years old, with a mean age at diagnosis of 65.0 ± 13.5 years, mainly affecting patients over 60 years (61.8%; 616 cases).

The most common site-affected subsite was the lower lip (79.4%; 793 cases), followed by lip, not otherwise specified (NOS; 9.5%; 95 cases) and upper lip (7.5%; 75 cases; Supplement 1). Most patients presented early-stage tumours (stages I-II) at diagnosis (83.3%; 810 cases). For most patients, the treatment was performed 60 days after diagnosis (69.5%; 423 cases), with surgery being the main treatment modality (72.0%; 718 cases), followed by radiotherapy (RT) alone (7.1%; 71 cases), and a combination of surgery and RT (7%; 69 cases). Approximately 5.1% (51 cases) of cases did not receive any active treatment, and the main reason was not specified (2.8%; 29 cases).

**Table 1:**
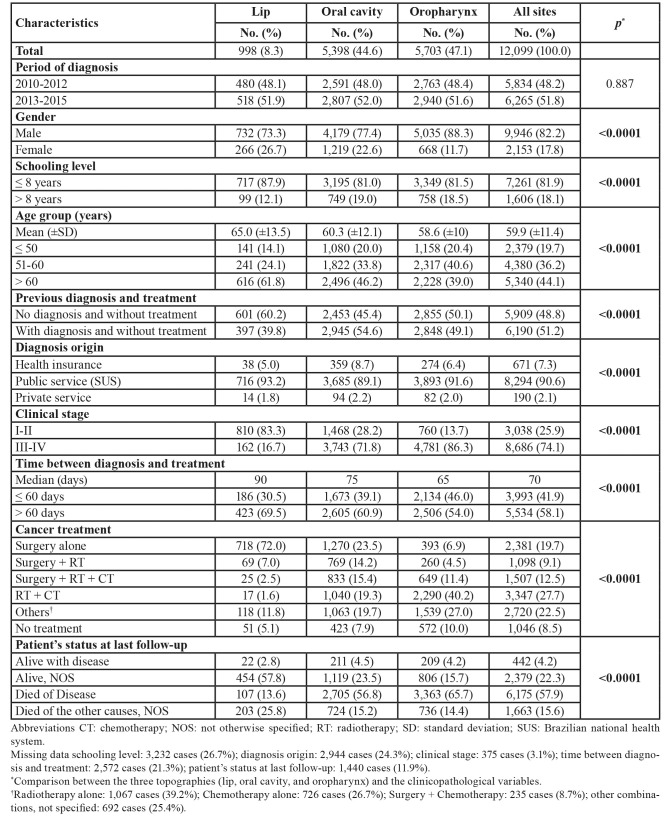
Demographical and clinicopathological features of 12,099 patients with lip, oral cavity, and oropharyngeal squamous cell carcinoma diagnosed in São Paulo State, 2010-2015.

**Figure 3:**
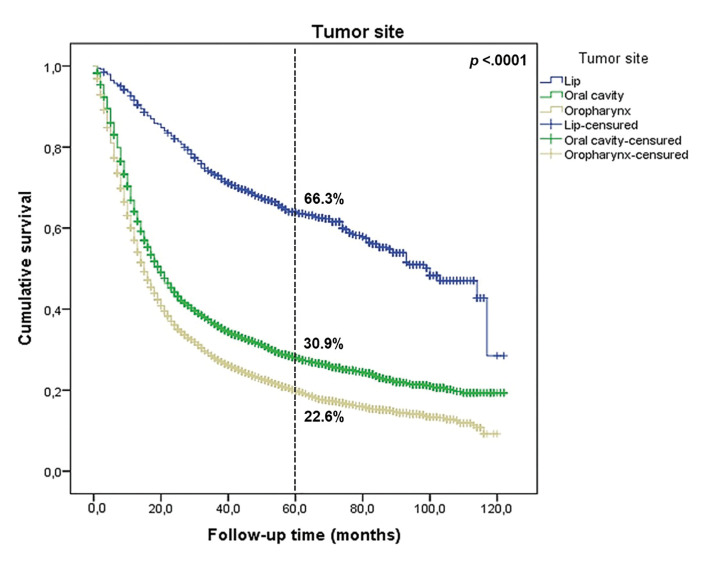
Five-year overall survival of 10,659 patients with lip, oral cavity, and oropharyngeal squamous cell carcinoma diagnosed in São Paulo State, 2010-2015, by tumour site.

Data from 789 (78.8%) individuals with lip SCC with a median follow-up time of 52 months were available (range: 1-122 months). Survival analysis from the Kaplan-Meier method estimated that the 5-year overall survival (OS) for lip SCC was 66.3% (Fig. 3). Based on the log-rank test (Supplement 2, Supplement 3 and Supplement 4), there was a significant increase in the OS for patients diagnosed in more recent years (58.7% in 2010-2012 to 72.7% in 2013-2015; *p* < 0.0001).

- Oral cavity SCC

Among 5,398 individuals with oral cavity SCC (OSCC), 77.4% (4,179 cases) were male, with a male-to-female ratio of 3.4:1. The patients’ ages ranged from 11 to 100 years old, with a mean age of 60.3 ± 12.1 years. The most affected age group were patients over 60 years old (46.2%; 2,496 cases). For schooling level, 81.0% (3,195 cases) of patients had less than or equal to 8 years of formal education.

The mobile tongue comprised 42.5% (2,298 cases) of cases, followed by the floor of the mouth (22.2%; 1,200 cases), mouth NOS (10.9%; 590 cases), retromolar trigone (7.9%; 429 cases), and hard palate (7.1%; 383 cases; Supplement 1). At diagnosis, 71.8% (3,743 cases) of patients were diagnosed with stages III/IV.

Approximately 60.9% (2,605 cases) of patients received treatment in the period of more than 60 days after diagnosis. Proportionally, surgery alone was the main treatment employed, being used in 23.5% (1,270 cases) of cases, followed by chemoradiotherapy (CT; 19.3%; 1,040 cases), and a combination of surgery, RT, and CT (15.4%; 833 cases). About 7.9% (423 cases) of individuals did not receive any treatment, and the main described reasons were that the patient died of disease before commencing treatment (3.1%; 172 cases) or had advanced untreaTable disease (1.8%; 98 cases).

Of those evaluated, 4,759 (88.2%) had a median follow-up time of 19 months (range: 1-122 months). The OS rate for OSCC was 30.9% in the 5 years after diagnosis (Fig. 1). However, an improvement was observed in the OS for patients diagnosed in the more recent years of study (25.1% in 2010-2012 to 35.8% in 2013-2015; *p* < 0.0001; Supplement 2, Supplement 5 and Supplement 6).

- Oropharyngeal SCC

Of the 5,703 patients with oropharyngeal SCC (OPSCC), 88.3% (5,035 cases) were male, with a male-to-female ratio of 7.5:1. The patients' ages ranged from 20 to 99 years old, with a mean age of 58.6 ± 10 years at diagnosis, with the most cases occurring in the sixth decade of life (40.6%; 2,317 cases). Based on schooling level, 81.5% (3,349 cases) of patients had less than or equal to 8 years of formal education.

For most cases the exact location of the tumour was not reported (oropharynx, NOS; 30.5%; 1,741 cases; Supplement 1). The base of the tongue (30%; 1,711 cases), tonsils (18.7%; 1,067 cases), soft palate (11.4%; 650 cases), and lateral oropharyngeal wall (3.2%; 180 cases) were the most affected sites of the oropharynx. Most of the tumours (86.3%) were at an advanced clinical stage (stages III-IV).

In most cases, the treatment was performed 60 days after diagnosis (54%; 2,506 cases), and chemoradiotherapy was the main treatment (40.2%; 2,290 cases), followed by a combination of surgery, RT, and CT (11.4%; 649 cases), and RT alone (10.2%; 579 cases). Approximately 10% (572 cases) of individuals did not receive any active treatment, and the main reasons reported were that the patient died of disease before commencing treatment 4.4% (258 cases) or had an advanced untreaTable tumour 2.6% (149 cases).

For survival analysis, 5,114 (89.7%) patients with a median follow-up time of 15 months were considered (range: 1-120 months). Five-year OS for OPSCC was 22.6% (Fig. 3), with a slight increase observed from 2013 to 2015, compared with the period of 2010 to 2012 (27.7% vs 20.2%, respectively; *p* < 0.0001; Supplement 2, Supplement 7 and Supplement 8).

The proportion of patients lost to follow-up was 21.2, 11.8, and 10.3% for lip SCC, OSCC, and OPSCC, respectively. Table 2 shows the variables that impacted the survival rate of the patients according to univariate Cox regression analysis. For lip SCC, the multivariate analysis model (Table 3) revealed that patients aged over 60 years (hazard ratio (HR): 2.45; 95% CI, 1.33-4.52), advanced-stage disease (HR: 1.97; 95% CI, 1.32-2.95), patients treated by chemoradiation (HR: 4.56; 95% CI, 2.15-9.67), and other treatments such as RT alone, CT alone, and other combinations (HR: 2.90; 95% CI, 2.06-4.08) were associated with a higher mortality hazard.

**Table 2:**
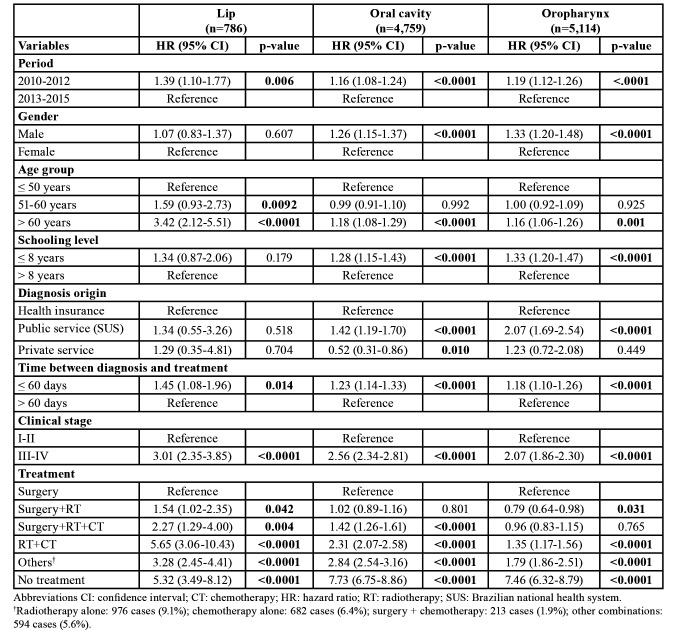
Relationship of demographics and clinicopathological variables to the hazard of death for patients with lip, oral cavity, and oropharyngeal squamous cell carcinoma diagnosed in São Paulo State, 2010-2015—univariate Cox regression.

**Table 3:**
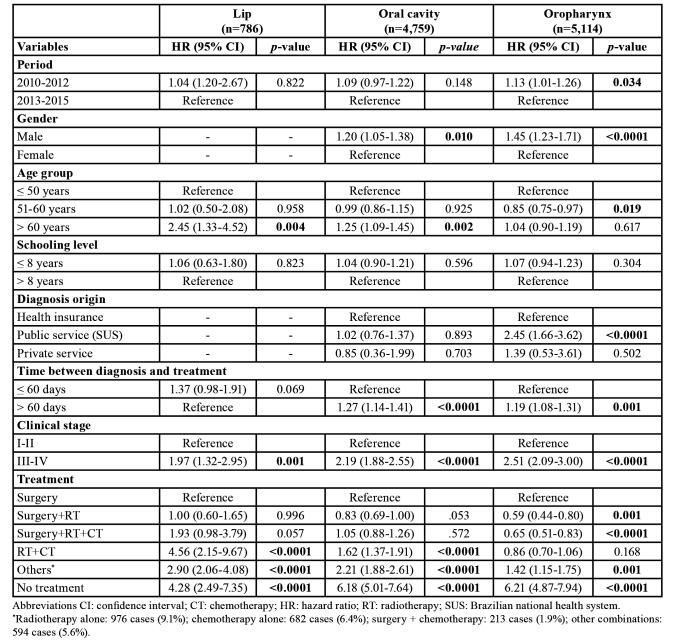
Relationship of demographics and clinicopathological variables to the hazard of death for patients with lip, oral cavity, and oropharyngeal squamous cell carcinoma diagnosed in São Paulo State, 2010-2015—multivariate Cox regression model created using all variables that achieved a *p-value* < 0.20 in univariate Cox regression analysis.

For patients with OSCC, mortality hazards were significantly higher among male patients (HR: 1.20; 95% CI, 1.05-1.38), in those over 60 years old (1.25; 95% CI, 1.09-1.45), with time between diagnosis and treatment over 60 days (HR: 1.27; 95% CI, 1.14-1.41), IN those with advanced-stage tumours (HR: 2.19; 95% CI, 1.88-2.55), and in patients treated by chemoradiation (HR: 1.62; 95% CI, 1.37-1.91) and other treatments (HR: 2.21; 95% CI, 1.88-2.61).

In OPSCC (Table 3), an increase in mortality hazard was observed for patients diagnosed between 2010-2012 (HR: 1.13; 95% CI, 1.01-1.26), in male individuals (HR: 1.45; 95% CI, 1.23-1.71), those with diagnosis from national public health system (SUS; HR: 2.45; 95% CI, 1.66-3.62), those with time between diagnosis and treatment over 60 days (HR: 1.19; 95% CI, 1.08-1.31), those with advanced stage cancer (HR: 2.51; 95% CI, 2.09-3.00), and patients treated by other treatments (HR: 1.42; 95% CI, 1.15-1.75).

## Discussion

Lip, oral cavity, and oropharyngeal cancers represent a major health problem in the global scenario, and together, comprise the eighth most common malignancy worldwide. Brazil has the highest incidence of oral and oropharyngeal cancer in Central and South America, and over 90% of cases are represented by the SCC (5).

Data retrieved from the FOSP showed that lip SCC, OSCC, and OPSCC accounted for 8.3 (998 cases), 44.6 (5,398 cases), and 47.1% (5,705 cases) of the cases evaluated, respectively, in 2010-2015. Among them, there was marked male predominance at the three sites, especially in the oropharynx, in accordance with the available literature (6-12). Chatuverdi *et al*. (3) reported a trend towards stabilization and even a slight decline in the incidence of OSCC and a significantly increased of OPSCC cases, mainly HPV-positive OPSCC, in middle- and high-income countries in the past decades, including Brazil. The high rate of OPSCC found in our study may reflect this increase observed in these tumours in the recent decades. However, the FOSP database did not report the HPV status in the recorded OPSCC cases, which makes the association between HPV infection and the high number of cases of OPSCC difficult. Recent studies from Brazil shows that the prevalence of HPV-positive OPSCC ranges from 6.1% to 59.1% (4,13).

Lip SCC and OSCC mainly occurred in older people. The average age at the time of diagnosis was approximately 65 ± 13.5 years and 60.3 ± 12.1 years in the present study, respectively, which corroborates previous studies performed in Italy (10), Mexico (14), and the United States (US) (15) for lip SCC and Brazil (8), Japan (16), and Australia (17) for OSCC. In contrast, the mean age at diagnosis was lowest for OPSCC (58.6 ± 10 years), with the prevalence peaking in the sixth decade of life. Similar findings were reported by other studies (6,18), in which the average age was lower compared to lip SCC and OSCC, mainly in the cases of HPV-positive OPSCC, where the mean age was usually less than 60 years (19,20).

Oral cancer is related to socioeconomic status and deprivation, with the highest incidence rates occurring in the most disadvantaged population groups. Moro *et al*. (9), Oliveira *et al*. (21), and Asio *et al*. (22) reported a marked association between lip SCC/OSCC/OPSCC and the low schooling level of patients. Similarly, 81.9% of all patients in the present study had up to 8 years of formal education. Nevertheless, studies from developed countries, such as the US (19) and Australia (12), reported higher educational levels in these patients.

The definition of the limits of the oral cavity varies between studies. Some authors include lips (9,14,22), whereas others do not (8,21). Due to this controversy, the lip and oral cavity were classified as different sites in this study. Lip SCC accounted for approximately one-third of OSCC cases (15). When lip SCC was exclusively analysed, previous studies reported that the lower lip was the most commonly affected site (10,15,23), similar to our findings. In the oral cavity, according to previous reports (7,9,14,22) and our results, the tongue (excluding the base of the tongue) was the most commonly affected subsite. However, in India and surrounding countries, the most frequent subsite of OSCC was the buccal mucosa, as a repercussion of the habit of chewing tobacco (24). Elwood *et al*. (18) and Dahlstrom *et al*. (19) reported that the most common subsite for OPSCC were tonsils, which is in contrast with the present study, where the base of the tongue was the most common subsite.

In general, the lip region is more accessible, facilitating early cancer detection and diagnosis (9). Previous studies performed in the US (15) and Serbia (23) reported that most lip SCC cases were in the early stage (stages I-II) at diagnosis, with few patients presenting regional and distant metastasis. In contrast, Fukumoto *et al*. (16), Oliveira *et al*. (21), and Listl *et al*. (25) described that the diagnosis of OSCC was usually delayed, allowing for local extension and regional metastasis; consequently, most cases were advanced-stage disease (stages III-IV). Schroeder *et al*. (20) and Kowalski *et al*. (8) observed that more than 70% of OPSCC patients were at stages III-IV. In agreement, these observations were consistent with our findings for the three sites.

Due to the early stages at the time of diagnosis, surgical resection with wide local excision is the main choice of treatment for lip SCC (15,23). Likewise, in our sample, 72% of lip SCC cases were treated with surgery alone. Although most cases were in the advanced stage, surgery alone was the most frequently employed treatment for OSCC cases in our sample, which corroborates previous reports (8,16,26). Nevertheless, in the studies performed by Asio *et al*. (22) and Oliveira *et al*. (21), RT alone was the most used treatment in OSCC cases. The oropharynx is not easy to access, and OPSCC usually presents as an advanced disease. Chemoradiotherapy was the main choice of treatment, being employed in approximately 40.2% of our cases, and confirming previous reports from Brazil (8) and another from the United Kingdom (20).

It is important to emphasise that lip SCC exhibited a better survival curve in our study, with a 5-year OS rate of 66.3%, which agreed with reports in the US (15) and Germany (25) that showed 5-year OS rates of 69.9 and 86.5%, respectively. Although advances in cancer treatments have occurred in recent decades, OSCC and OPSCC are still considered cancers with poor prognosis, presenting lower survival rates when compared to lip SCC. The SEER database analysis by Farhood *et al*. (27) demonstrated an OS rate of 49% at 5 years after the initial diagnosis for OSCC. A study conducted in Northeast China (28) found that the 5-year OS rate was slightly better than the report from the US, at 54.5%. The worst outcomes were reported in southern Taiwan (29) and Uganda (22), in which the 5-year OS rates were 36.1 and 20.7%, respectively. Similarly, a 5-year OS rate of 30.9% for OSCC was observed in the current study.

Tumours located in the oropharynx present worse survival rates, especially in HPV-negative cases (1). OPSCC showed a lower 5-year OS (22.6%) between the three sites analysed in the sample. Similarly, Miller *et al*. (30) reported 5-year OS of 29.6%. A study conducted by Fakhry *et al*. (31) concluded that when compared to p16-negative OPSCC patients, p16-positive OPSCC patients had an estimated 52% reduction in risk of death being associated with better OS rates (20). Similarly, Abrahão *et al*. (1) found that 3-year OS rates were 44.6% and 75.6% for p16-negative OPSCC and p16-positive OPSCC, respectively, and concluded that HPV status was an important prognosis predictor of OS (HR: 3.35; 95% CI, 1.33-8.45). Although the FOSP database did not provide HPV status, we hypothesised that low OS may be associated with a high prevalence of HPV-negative OPSCC in our sample, which would agree with similar studies where a lower survival was observed for those with HPV-negative tumours (20,32). Another possibility is that, although the majority are HPV-positive, most patients report being smokers, and thus, have a prognosis like those with HPV-negative tumours, as reported by another study carried out in a Brazilian cancer center that founded those patients with HPV-positive OPSCC and with previous history of heavy use of tobacco was not associated with higher survival rates (4).

Male sex was an independent predictor of OS in the multivariate analysis for OSCC (HR: 1.20; 95% CI, 1.05-1.38) and OPSCC (HR: 1.45; 95% CI, 1.23-1.71). These findings were consistent with a study that collected data from four countries in South America, in which male patients with OPSCC (HR: 1.84; 95% CI, 1.08-3.14) (1) presented higher mortality rates than females. Nevertheless, Farhood *et al*. (27) (HR: 0.98; 95% CI, 0.93-1.04) and Kowalski *et al*. (8) (HR: 1.14; 95% CI, 0.86-1.51) did not observe an increase in mortality hazard for male patients with OSCC. In contrast, this study showed that the increasing age for patients with lip SCC (> 60 years—HR: 2.45; 95% CI, 1.33-4.52) and OSCC (> 60 years—HR: 1.25; 95% CI, 1.09-1.45) was associated with low OS rates, which corroborates the findings by Han *et al*. (15) (HR, 1.07; 95% CI, 1.07-1.08) for lip SCC and Abrahão *et al*. (1) (HR: 1.82; 95% CI, 1.18-2.78) for OSCC. Individuals diagnosed with lip SCC (HR: 1.97; 95% CI, 1.32-2.95), OSCC (HR: 2.19; 95% CI, 1.88-2.55), and OPSCC (HR: 2.51; 95% CI, 2.09-3.00) with advanced-stage (stages III-IV) tumours were more likely to die than patients with early-stage disease, which was an important independent determinant of OS, corroborating the findings reported in earlier studies (7,8,15,22,27).

Pathology laboratories provide cancer diagnostic services and key prognostic factors that guide patient treatment decision. In Brazil, the university oral pathology laboratories performed an important role in oral cancer diagnosis and the national public health system (SUS) (33). In our study, patients with OPSCC diagnosed by public laboratories/hospitals (SUS) presented higher mortality rates (HR: 2.45; 95% CI, 1.66-3.62). However, these findings for patients with OPSCC can be attributed to several reasons and may include lack of awareness of cancer signs and symptoms, the patient, who can often take a long time to seek care, and the access to the public health service for the biopsy which can be difficult, especially in public tertiary health centers, including oncology hospitals. Therefore, most cases are diagnosed in advanced-stages and, consequently, the potential curability of OPSCC decreases considerably (1,34).

The delay between diagnosis and the start of treatment at over 60 days was associated with a high mortality hazard for OSCC (HR: 1.27; 95% CI, 1.14-1.41) and OPSCC (HD: 1.19; 95% CI, 1.08-1.31) patients. In Australia (17), the median time between diagnosis and treatment was 30 days for OSCC, and in Brazil, the median time was up to 3-times higher (34), which was similar to our findings (75 days). Finally, according to Felippu *et al*. (34), this delay was associated with factors such as the low intellectual and social status of most patients, as well as the shortcomings of the public health care system.

Patients treated with surgery alone presented higher survival rates compared to patients treated with combinations of RT and CT. Fukumoto *et al*. (16), Bai *et al*. (7), and Farhood *et al*. (27) found similar results. However, the treatment must be done carefully, as advanced-stage disease usually requires more complex treatments with the use of RT and/or CT. Furthermore, the protocols used and the patient's collaboration can also influence the choice of treatment.

## Conclusions

Based on this robust analysis of 12,099 cases of lip SCC, OSCC, and OPSCC derived from the FOSP database, this report highlights a marked male predominance, mainly affecting patients over 60 years old and with less than or equal to 8 years of education, presenting as an advanced-stage (stages III-IV) disease. The independent prognostic factors varied according to tumour site in multivariate analysis, except for tumour stage, which was a significant determinant of survival for all three sites. In addition, OSCC and OPSCC presented worse 5-year OS rates, whereas lip SCC had a high OS rate. However, an improvement in OS was observed for patients diagnosed in the more recent years of study (2013-2015).
